# Evaluation of fruit combinations as potential liquid attractants for hydrogel bait applications targeting *Aedes* mosquitoes

**DOI:** 10.1186/s13071-025-07204-0

**Published:** 2026-01-05

**Authors:** Noor Muokhni Ayub, Nur Faeza Abu Kassim, Sumiyyah Sabar, Nur Aida Hashim, Japareng Lalung, Shaida Fariza Sulaiman, Sara A. Abuelmaali, Cameron E. Webb

**Affiliations:** 1 129 Medical Entomology Laboratory, School of Biological Sciences, Universiti Sains Malaysia, 11800 Minden, Penang Malaysia; 2 Chemical Sciences Programme, School of Distance Education (SDE), Universiti Sains Malaysia, 11800 Minden, Penang Malaysia; 3 Faculty of Food Science and Agrotechnology, Universiti Malaysia Terengganu, AFF3 - 21030 Terengganu, Malaysia; 4 School of Technology Industry, Universiti Sains Malaysia, 11800 Minden, Penang Malaysia; 5 Medical Entomology, NSW Health Pathology, Westmead Hospital, Westmead, NSW 2145 Australia; 6 Sydney Institute for Infectious Diseases, University of Sydney, Westmead Institute for Medical Research, 176 Hawkesbury Road, Westmead, NSW 2145 Australia; 7 Department of medical entomology, National public health alboratory/Federal ministry of health, Khartoum, Australia

**Keywords:** Attractive toxic sugar bait (ATSB), *Aedes aegypti*, *Aedes albopictus*, Mango, Banana

## Abstract

**Background:**

A major challenge to global vector control efforts is the increasing resistance of *Aedes* mosquitoes to conventional insecticides. Since they are the main vectors of dengue, Zika, and chikungunya, sustainable and environmentally friendly approaches are essential for vector control. Attractive toxic sugar baits (ATSBs) take advantage of mosquitoes’ propensity for sugar and can offer an alternative strategy. However, further research is needed to investigate the performance of ATSBs, especially in determining and assessing attractant combinations that might increase mosquito attraction and feeding efficiency.

**Methods:**

This study examines the feeding preferences of *Ae. aegypti* and *Ae. albopictus* for several fruit-based ATSB formulations. We employed three assays of attractants: mango alone, banana alone, and a combined mango–banana formulation, as well as a control set. Three replicates of each species at a 50% dosage of each fruit extract were evaluated.

**Results:**

The findings indicate that combining fruit sources enhances mosquito attraction, since there is a statistically significant preference for the mixed fruit formulation (1:1) compared with the single-fruit attractants (*P* < 0.05). However, there were no significant differences in the feeding preferences between the males and the females, indicating that the treatment effect is equal for both genders.

**Conclusions:**

This study contributes to the ongoing advancement of sustainable and efficient vector control strategies by demonstrating the enhanced attractiveness of mixed-fruit formulations, which offer environmentally safe methods for managing *Aedes* mosquito vectors and arboviral diseases.

**Graphical abstract:**

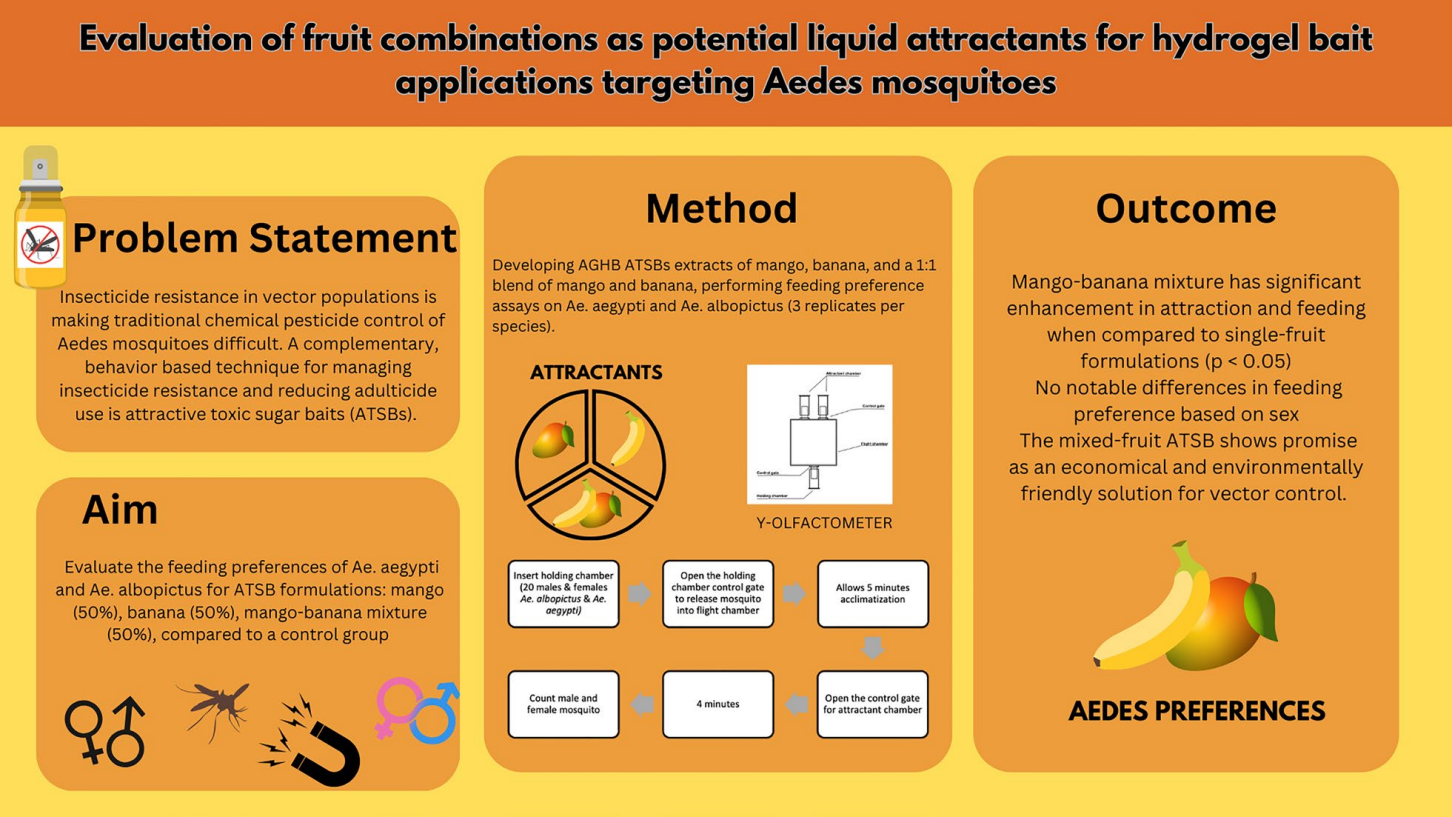

**Supplementary Information:**

The online version contains supplementary material available at 10.1186/s13071-025-07204-0.

## Background

Mosquito-borne diseases have resulted in the deaths of more than 1.5 million individuals annually on a global scale [1]. The World Health Organization (WHO) reports 100–400 million infections annually, with the majority exhibiting asymptomatic or moderate symptoms, and over 3.9 billion people are susceptible to dengue viruses. Dengue viruses kill between 10,000 and 20,000 people each year [1, 2]. Vector-borne diseases are a critical issue for human health, specifically those vectored and transmitted by *Aedes* spp., including chikungunya, Zika, yellow fever, and dengue viruses [3, 4]. The incidence of the diseases caused by *Aedes* mosquitoes has increased and expanded into new geographical areas, which correlates with increasing population density, international travel, and the import and export of goods [5, 6]. Since vaccines are often unavailable, the best way to prevent epidemics is by controlling the mosquito vector [7].

One promising approach to *Aedes* mosquito control is attractive toxic sugar bait (ATSB), which exploits their natural sugar-feeding behavior [8, 9]. ATSBs incorporate attractive components, such as sucrose, nectar, and fruit-based volatiles, to attract mosquitoes toward bait containing insecticide. Previous studies have demonstrated ATSB effectiveness in reducing mosquito populations, highlighting their potential as an essential vector control tool [10]. Fruit-based attractants are particularly promising due to their rich sugar content and volatile compounds. Studies have proven that *Aedes* mosquitoes are highly attracted to subtropical fruits such as guava, mango, and banana [11]. Mango (*Mangifera indica* L.), a member of the Anacardiaceous family, is well known for its high sugar content and volatile chemicals such as aldehydes, monoterpenes, and sesquiterpenes, which have been shown to attract mosquitoes. These molecules add to the fruit’s balm and flavor, and some of them are also attractive to insects, such as mosquitoes [12].

Similarly, banana (*Musa acuminata*) emits octanol, a compound that has also been linked to the attraction of mosquitoes [9]. Cavendish banana, a subgroup of the AAA group, contains volatile compounds, such as acetates, butanoates, and 3-methyl butyl ester, which may enhance mosquito response [13]. While limited research has focused on the combined effect of mango and banana as mosquito attractants, present studies suggest a positive attraction to both fruits. ATSB formulations with guava, mango, and cupuaçu combined with boric acid have been shown to cause significant mortality in *Ae. aegypti* males and females [9]. Similarly, mango-based ATSBs with pyriproxyfen effectively inhibited mosquitoes’ emergence. Mosquitoes are attracted to sugar sources through volatile semiochemicals, detected by olfactory receptors on their antennae. Gas chromatography–mass spectrometry (GC–MS) analysis has identified 23 volatile compounds in mango, including aldehydes, alkanes, benzenoids, monoterpenes, sesquiterpenes, and oxygenated terpenes [8]. Recent research on how mosquitoes smell has shown that *Ae. aegypti* mosquitoes are very sensitive to a wide range of volatile organic compounds (VOCs), many of which are chemically similar to those released by ripe fruits. Banana volatiles such as butanoic acid, 3-methyl butyl ester, 1-butanol acetate, acetic acid hexyl ester, hexanal, and 1-hexanol are known to attract other dipteran insects, such as fruit flies, because of the way they break down. These chemicals resemble human skin odors, which mosquitoes use to find hosts [14]. While direct proof of *Ae. aegypti* attraction to banana volatiles is limited, the similarity in their chemical structures implies a potential involvement in mosquito attraction, necessitating more investigation [14–16].

The controlled release of insect growth regulators (IGRs) and other chemical pesticides has historically been accomplished in pest management using synthetic hydrogels such as polyacrylamide and talc-based gels [17, 18]. However, their incompatibility with semiochemical-based solutions, potential issues with pesticide release, and the dispersion of nonbiodegradable components raise environmental concerns [17]. Hydrogel polymers have shown promising potential in mosquito control, especially as carriers for genetic materials and insecticides [19]. In biodistribution studies, hydrogels have been investigated not only as bait carriers but also as nanoparticles (NPs) for the delivery of nucleic-acid-based mosquitocidal molecules [19], and genetic materials such as double-stranded RNA (dsRNA) and small interfering RNA (siRNA) to mosquito tissues [20].

The use of biodegradable hydrogels to more precisely target mosquito larvae is the subject of recent advancements. For instance, *Bacillus thuringiensis israelensis* (Bti) microencapsulation in alginate has been demonstrated to protect the bacteria from ultraviolet degradation and improve its slow release in field conditions [21]. Alginate–chitosan polymers have also been developed as carriers for cinnamaldehyde in controlling *Ae. aegypti* larvae [22]. Furthermore, *Anopheles gambiae* larvae have been shown to be more susceptible to pesticides such as diflubenzuron when their chitin synthase genes are silenced by chitosan/dsRNA self-assembled nanoparticles [21]. These experiments showed how hydrogel-based, eco-friendly mosquito control methods can be used as part of integrated vector management plans in the future.

Despite increasing interest in attractive toxic sugar baits (ATSBs), there is still a limited understanding of the impact of specific fruit-derived attractants and their combinations on mosquito attraction and feeding behavior. The potential synergistic effects of combining various fruit volatiles remain inadequately investigated. This study investigates a mango–banana mixture as a potential attractant for alginate–gelatin hydrogel bead (AGHB) bait. The volatile profiles of mangoes and bananas are distinct, and their combination may improve the overall attractiveness and feeding response of *Aedes* mosquitoes. We characterized the volatile compounds in the mixture using headspace GC–MS. Comprehending these interactions could facilitate the creation of a cost-efficient, environmentally sustainable ATSB formulation that enhances mosquito control efficacy.

## Methods

### Mosquito culture

Laboratory egg strains of *Aedes aegypti* and *Ae. albopictus *were obtained from the Vector Control and Research Unit (VCRU), Universiti Sains Malaysia (USM). The mosquitoes were reared and maintained in insectary G25-B, School of Biological Sciences, USM at 27 °C, with 80% relative humidity and 14-h:10-h light–dark cycle [23]. The eggs were submerged in a container filled with dechlorinated water, while the emerged larvae were then fed with larvae food prepared with a blend of cat food, beef liver, yeast, and milk powder (2:1:1:1). Food materials were given frequently, and the water container was changed every 2 days. The pupae were transferred into plastic cups and placed in the rearing cages (30 cm × 30 cm × 30 cm, length × breadth × height).

After emergence, adult mosquitoes were provided with a 10% sucrose solution and were blood-fed twice a week using mice supplied by the Animal Research and Service Centre (ARASC), USM. The mosquitoes starved for 1 day prior to blood feeding. Then, a filter paper soaked with distilled water and placed in a Petri dish in the rearing cage for oviposition. The filter paper containing the eggs was allowed to air dry and then stored in a dry container.

### Fruit extraction

One kilogram of fresh mango (*Mangifera indica* L.) and Cavendish banana from the subgroup of the AAA group (*Musa acuminata*) fruits were obtained from the Lotus’s store, Penang; the fruits were barely ripe. The fruits were washed with tap water and then peeled before being sliced into small pieces. Each fruit was minced and blended using a dry blender (Toshiba® BL-70PR1NMY, 700 W) without adding any water to obtain the pure fresh fruit pulp and fiber. The blended smoothies were filtered (double filtration) using industrial sieve equipment in the laboratory to remove pulp or seeds from the fruits (Fig. S1). The product was then treated with 2% w/v sodium benzoate, which acts as a preservative to increase its longevity.

### Preparation of AGHBs as a carrier for the liquid attractants

Alginate–gelatin hydrogel beads (AGHBs) were prepared following a previously described method [24]. Sodium alginate (Na-Alg) with a viscosity of 3500 cps (2% v/v at 25 °C) and gelatin from bovine skin (Type B, powder, bioreagent) were purchased from Sigma-Aldrich (St. Louis, MO, USA). To prepare the hydrogel beads, 8% alginate (w/v) and 1% gelatin (w/v) were dissolved in 100 mL of distilled water and mixed until fully homogenized. The resulting solution was added dropwise into a 0.2 M calcium chloride (CaCl_2_) solution using a 500 mL burrete with a 1-cm-diameter tip. The distance between the burette tip and the surface of CaCl_2_ solution was fixed to 8 cm while stirring for 5–10 min, leading to the instant formation of spherical beads (size range 0.5–1 cm). The beads were washed three times with distilled, filtered water [25]. To load the attractant, the produced hydrogel beads were soaked in fruit juice (single or mixed) for 24 h, allowing the optimal absorption of the attractant solution.

### *Aedes aegypti* bioassay

The mosquitoes’ response toward different fruit extracts was evaluated using a modified olfactometer cage [25, 26]. The cage was designed with dimensions of 15 cm × 15 cm × 15 cm (length × breadth × height) and was attached to two attractant chambers (diameter 4.4 cm, length 12.5 cm) at one end, as well as one holding chamber (diameter 4.4 cm, length 12.5 cm) that was detached from the flight cage. The holding chamber was attached to the flight cage during the release process. Chamber 1 (C1) and chamber 2 (C2) served as attractant chambers, each labeled with their respective attractants (Fig. S2). To minimize potential position bias, the chambers designated as C1 and C2 were used interchangeably during testing rather than being fixed to one side. Each chamber was cleaned with 70% denatured alcohol and rinsed three times between tests. No external airflow was applied, and both chambers were positioned under identical lighting conditions to ensure experimental symmetry.

A total of 40 adult *Ae. aegypti* (20 males and 20 females) were starved separately for 24 h in the holding chamber before the preference test. Then, the mosquitoes were released into the flight cage attached to the attractant chamber. The attractant chamber stayed closed during the initial release to allow the mosquitoes to acclimate to the cage. After 1 h of acclimation, the attractant chambers were opened. A fan was placed at the end of each mesh cap attractant chamber to allow the scent to flow into the cage. The mosquitoes were allowed to fly in the flight chambers, and after 4 min, the chamber gates were closed. The number of mosquitoes on the mesh screens was counted, sorted by sex, and removed from the flight cage. The test was replicated three times for each set of attractant pairs as listed in Table S1.

### *Aedes albopictus* bioassay

The bioassay was conducted following a previously established method [25, 26] using 20 adult males and females of *Ae. albopictus* per trial. Each test was replicated three times to ensure reliability. We repeated the same procedure mentioned above in the *Ae. aegypti* bioassay.

### Analysis of volatile chemical constituents

Volatile organic compounds (VOCs) from the attractants were analyzed using headspace solid-phase dynamic extraction gas chromatography/mass spectrometry (HS-SPDE-GC/MS) with a GC-2010 Plus model (Shimadzu Corp.). This analysis was employed to identify key chemical constituents responsible for mosquito attraction to AGHBs loaded with fruit-based attractants. For sample preparation, 10 mL of minced fruit was placed in a tightly sealed vial with a PTFE-silicon septum to prevent contamination. They were then heated at 40 °C for 2 min to facilitate the release of volatiles into the headspace. The extracted gas phase was injected into the GC–MS system for 1 min under a low shaker mode during sample heating [22]. This analysis allowed the precise identification of semiochemicals that influence mosquito behavior. The detected VOCs were compared against known mosquito attractants to determine their potential role in enhancing the efficacy of AGHBs in ATSB applications.

### Data analysis

The mean ± SE of the liquid-attractant concentrations was analyzed using one-way analysis of variance (ANOVA), followed by Tukey’s honestly significant difference (HSD) test for multiple comparisons. Statistical significance was set at *α* < 0.05 for all test sets. The attractancy effect of mosquitoes toward each attractant was evaluated using the preference index (PI). To meet the assumptions of *t*-test analysis, PI values were arcsine square-root transformed before applying Student’s *t*-test for pairwise comparisons. Results were illustrated using bar graphs, with error bars indicating standard error (SE). To assess mosquito distribution within the olfactometer chambers, an exact binomial test was conducted to determine whether mosquito choice deviated from the expected 50:50 ratio under the null hypothesis. Differences between male and female mosquito responses were analyzed on the basis of PI values, which range from −1 to +1, with positive values (+1 to 0) indicating attraction to the test stimulus, and negative values (0 to −1) indicating repellency or avoidance. A PI of 0 indicates a neutral response with no preference for either attractant. The PI was calculated using the following equation:

$$\mathrm{Preference} \mathrm{Index} (\mathrm{PI})=\frac{\mathrm{Number} \mathrm{of} \mathrm{mosquitoes} \mathrm{in} \mathrm{Chamber} 1 -\mathrm{Number} \mathrm{of} \mathrm{mosquitoes} \mathrm{in} \mathrm{Chamber} 2}{\mathrm{Number} \mathrm{of} \mathrm{mosquitoes} \mathrm{in} \mathrm{Chamber} 1+\mathrm{Number} \mathrm{of} \mathrm{mosquitoes} \mathrm{in} \mathrm{Chamber} 2}$$

## Results

### Response of *Ae. aegypti* to the different sets of attractants:

The PI of *Ae. aegypti* was evaluated on the basis of their response to five sets of attractant pairs. The bar graphs in Fig. S3 illustrate the attractancy (positive response) and repellency (negative response) of mosquitoes toward the tested attractants. For set A (Fig. S3 (a); mango versus sucrose), *Ae. aegypti* showed a significant preference for mango compared to sucrose (*t*(3) = 8.71, *P* = 0.00). The mean PI between sexes indicates that females (1.0 ± 0) showed a higher attraction to mango compared with males (0.48 ± 0.20) (Table S2). Similarly, for set B (Fig. S3 (b); banana versus sucrose), mosquitoes significantly preferred banana over sucrose (*t*(3) = 8.12, *P* = 0.00). The mean PI ± SE between sexes indicated that males (0.92 ± 0.08) showed a stronger preference for banana than females (0.5 ± 0.29) (Table S2).

For set C (Fig. S3 (c); mango versus banana), there was no statistically significant difference in the attraction of *Ae. aegypti* to mango and banana (*t*(6) = 0.17, *P* = 0.87). The mean PI ± SE indicated that males (0.14 ± 0.19) were more attracted to banana, while females (0.25 ± 0.43) showed a slight preference for mango (Table S2). For set D (Fig. S3 (d); mixed versus mango), mosquitoes displayed a significant preference for the mixed attractant over mango (*t*(3) = 8.94, *P* = 0.00). The mean PI ± SE between sexes showed that males (0.67 ± 0.19) mostly preferred the mixed attractant compared to females (0.30 ± 0.24) (Table S2). Lastly, for set E (Fig. S3 (e); mixed and banana), *Ae. aegypti* showed a significant preference for the mixed attractant over banana (*t*(3) = 4.42, *P* = 0.02).

In the comparison of sexes (Table S2), the mean PI ± SE indicated that *Aedes* males (0.43 ± 0.25) exhibited a greater attraction to the mixed attractant than females (0.38 ± 0.24).

*Aedes aegypti* males exhibited an increased preference for the mixed and banana attractant in contrast to mango, whereas females showed a stronger attraction to the mixed or mango attractant relative to banana. The combined attractant was the most favored by both males and females.

### Response of *Ae. albopictus* to the different sets of attractants:

The results for set A (Fig. S4 (a); mango versus sucrose) indicated that *Ae. albopictus* exhibited a substantial preference for mango over sucrose (*t*(3) = 5.64, *P* = 0.01). The average PI across sexes (Table S3) revealed that females (0.88 ± 0.13) exhibited a greater attraction to mango than males (0.58 ± 0.25). In set B (Fig. S4 (b); banana versus sucrose), the mosquitoes exhibited a statistically significant preference for banana over sucrose (*t*(3) = 7.84, *P* = 0.01). Males (0.88 ± 0.13) had a greater attraction than females (0.38 ± 0.24). In set C (Fig. S4 (c); mango versus banana), no statistically significant difference was seen in the attraction of *Ae. albopictus* to mango and banana (*t*(3) = 2.93, *P* = 0.06). In set D (Fig. S4 (d); mixed versus mango), mosquitoes exhibited a substantially greater attraction to the mixed attractant than to mango (*t*(3) = 4.69, *P* = 0.02). Males (0.75 ± 0.25) exhibited a greater preference for the mixed attractant than females (0.53 ± 0.18). In set E (Fig. S4 (e); mixed versus banana), mosquitoes showed a pronounced preference for the mixed attractant over banana (*t*(3) = 5.72, *P* = 0.01). Females (0.58 ± 0.25) displayed greater attraction to the mixed attractant than males (0.25 ± 0.24).

### Headspace gas chromatography–mass spectrometry analysis

A headspace gas chromatography–mass spectrometry (GC–MS) analysis of the VOCs released from mango and banana attractants identified 12 distinct compounds (Table S4). The mango attractant primarily emitted monoterpenes (α-pinene, ß-pinene, and ß-ocimene) and sesquiterpenes (α-humulene, caryophyllene, and σ-cadinene), which are known to influence mosquito olfactory responses [27]. In addition, alcohols (Z)-3-hexen-1-ol and aldehydes (carbonyl compounds) were detected, both of which have been previously reported as semiochemicals affecting insect behavior [9]. By contrast, the banana attractant contained esters (ethyl acetate and isoamyl acetate) and carboxylic acids (propanoic acid, 2-methyl, and hexanoic acid), which are associated with fruit fermentation and known to attract various insect species [9]. Notably, hexanol was present in both mango and banana samples, suggesting its potential role as a shared attractant.

## Discussion

### Fruit preference in mosquito feeding behavior

Mosquitoes require sugar meals as their primary carbohydrate source, which is essential for maintaining energy levels and body fitness. As adaptable sugar feeders, they readily consumed available sugar sources that provide sufficient energy [28]. Phytophagy, the ability of mosquitoes to obtain nutrients from plant-based sources, includes the ingestion of nectar (nectarivore), fruit juices (frugivory), plant sap, and extrudes [13]. Given this natural feeding behavior, developing bait attractants that mimic natural sugar sources could enhance the effectiveness of mosquito control strategies by competing with naturally available sugar sources in the environment.

Sugar selection in mosquitoes may be influenced by species-specific feeding behavior, including exophagic (outdoor feeding) and endophagic (indoor feeding) tendencies [29, 30]. The data collected in this study indicate that both *Ae. aegypti* and *Ae. albopictus* were equally attracted to both fruits (mango and banana) when given a choice. Since mosquitoes rely on phytophagy to meet their carbohydrate needs, the chemical volatiles emitted by mango and banana appear to play a role in attracting *Aedes* mosquitoes [31]. The results of this study confirm that fruit extracts elicit a stronger attraction compared with glucose, supporting the conclusion that fruit-based attractants are a vital component in the development of AGHBs baiting tools.

While glucose alone functions as a phagostimulant for bait formulations, the incorporation of fruit-based attractants significantly enhances the attractancy of AGHBs. Increasing bait effectiveness is essential to compete with naturally available sugar sources in mosquito habitats. The concept of blending fruit attractants is particularly promising to improve the efficacy of ATSBs. In this study, a 1:1 mixture of equal amounts of mixed banana and mango extract was used as a blended attractant. Mosquitoes were exposed simultaneously to mixed and single fruit attractants (banana or mango) to assess their preference.

The findings suggested that combining fruit volatiles enhances mosquito attraction and may provide a novel approach for improving ATSBs in vector control programs. Mango and banana are well-established attractants in ATSB applications, and previous research has shown that juices from subtropical fruits such as guava, mango, and banana attract *Anopheles* spp. [18, 23, 32]. These results further justify the potential of fruit-based attractants in mosquito control strategies.

Although other studies have explored blended fruit attractants for bait traps [9, 21], most research has focused on trapping strategies rather than the direct application of mixed-fruit attractants. For instance, a passive trap for collecting *Ae. aegypti* males used a mango–guava mixture to attract mosquitoes as part of the sterile insect technique (SIT) program [33]. However, this study primarily addressed SIT-based trapping rather than evaluating the effects of mixed fruit attractants on mosquito behavior. Further research is needed to explore different fruit combinations and their potential to optimize ATSB formulations. In addition, repetitive feeding attempts on AGHBs were observed 24 h after the first exposure, suggesting that mosquitoes did not develop aversive learning toward the bait. This behavioral response needs to be further investigated to determine its implications for long-term bait effectiveness and product development.

### Sex-based differences in mosquito response

Both male and female mosquitoes of *Ae. aegypti* and *Ae. albopictus* exhibited a strong preference for the mixed fruit attractant. However, when presented with mango and banana separately, males of both species showed a greater preference for banana over mango. This suggests that mango may lack certain nutrients essential for male reproductive development [34, 35]. Previous studies have shown that banana consumption enhances mating performance in *Anopheles coluzzii*, with males preferentially feeding on banana and mango juice after swarming activities [36]. By contrast, female *Ae. aegypti* and *Ae. albopictus* demonstrated a stronger preference for mango over banana [37]. Furthermore, because regular sugar meals are necessary for energy and nutrition, *Ae. albopictus* actively forages for sugar sources and is drawn to a variety of plants. Studies conducted in the laboratory and field have shown this behavior [38]. Notably, female *Ae. albopictus* cannot survive on a blood diet only [39, 40] because blood meals are primarily used for egg maturation rather than maintaining energy metabolism. In mosquito control strategies, dual ATSBs have been developed by incorporating mango-based attractants mixed with host kairomones to target female *Aedes* mosquitoes [25].

### Volatile components

Volatile compounds from attractants play a crucial role in developing effective hydrogel-based baiting systems. Certain floral semiochemicals act as olfactory cues, guiding mosquitoes to sugar sources. Many previous studies reported on ATSBs for mosquito control have used mango-based attractants, while banana has also been incorporated. However, our research suggests that combining mango and banana enhances better attractant efficacy, effectively targeting both *Ae. aegypti* and *Ae. albopictus*. This is due to the complementary nature of their volatile profiles, as each fruit emits distinct chemical classes, including alcohols, aldehydes, sesquiterpenes, monoterpenes, and carboxylic acids.

In mango-based attractants, *Aedes* mosquitoes primarily detect aldehydes, alcohols, monoterpenes, and sesquiterpenes. Monoterpenes and sesquiterpenes contribute to the flavor and aroma profile of the fruit. However, sesquiterpenes are less volatile than monoterpenes. Specific monoterpenes detectable by *Aedes* include α-pinene, thujone, and β-ocimene, while sesquiterpenes such as α-humulene, caryophyllene, and σ-cadinene are also known as attractants. A pioneering study by Nyasembe [41] demonstrated the potential of these volatile compounds in attracting both male and female *Anopheles* mosquitoes. In addition, aldehydes such as hexanal and alcohols such as (*Z*) 3-hexen-1-ol play a significant role in fruit–mosquito interactions, acting as chemical cues that influence mosquito foraging and host-seeking behavior.

## Conclusions

Optimizing attractive components is crucial for designing effective baiting tools in mosquito vector control. This study demonstrates that both *Aedes aegypti* and *Ae. albopictus* exhibit species- and sex-specific preferences for fruit-based attractants. The study findings highlight the effectiveness of mixed-fruit formulations in enhancing mosquito attraction, making them more suitable than single-fruit attractants for ATSB applications. The mixed-fruit formulation’s increased appeal is likely due to the synergistic effects of volatile compounds from mango and banana, such as α-pinene, β-pinene, β-ocimene, α-humulene, caryophyllene, σ-cadinene, ethyl acetate, isoamyl acetate, propanoic acid, and hex. These chemicals enhance mosquito feeding by modifying olfactory signals to sugar sources. The controlled laboratory settings in which this study was carried out might not fully reflect the variety of environmental elements seen in real ecosystems. Because of the brief exposure period, it may have been more difficult to identify long-term behavioral changes or differences in mosquito attractant preferences. Furthermore, the efficacy of the mixed fruit-based attractant was not assessed in the field; consequently, additional studies in natural settings are required to confirm its performance and practicality.

In addition, future studies should concentrate on determining the precise functions of distinct volatile chemicals in attracting mosquitoes, verifying laboratory results in the field, and enhancing the stability and effectiveness of bait formulations. Finally, developing attract-and-kill strategies for mosquito vector management will require investigating species-specific attractant blends, assessing their interactions with insecticidal chemicals, and evaluating potential effects on nontarget organisms.

## Supplementary Information

Additional file 1: Fig. S1. Procedure for preparation of fruits solution. Fig. S2. Schematic diagram of the modified Y-olfactometer used to evaluate *Aedes* mosquito responses to attractants. Fig. S3. Bar graphs showing the mean PI ± SE of *Aedes aegypti* (males and females) in response to five sets of attractants (a) mango versus sucrose, (b) banana versus sucrose, (c) mango versus banana, (d) mixed versus mango, and (e) mixed versus banana. Positive PI values indicate attraction, whereas negative PI values indicate repellency. Fig. S4. Bar graphs showing the mean PI ± SE of *Aedes albopictus* (males and females) in response to five sets of attractants (a) mango versus sucrose, (b) banana versus sucrose, (c) mango versus banana, (d) mixed versus mango, and (e) mixed versus banana. Positive PI values indicate attraction, whereas negative PI values indicate repellency.

Additional file 2: Table S1. Single and mixed attractants for mosquito’s preference tests. Table S2. Mean PI ± SE of male and female *Aedes aegypti* in response to five sets of attractants based. Table S3. Mean PI ± SE of male and female *Aedes albopictus* in response to five sets of attractants. Table S4. Volatile organic compounds (VOCs) identified in the mango and banana attractant mixtures.

## Data Availability

Data supporting the main conclusions of this study are included in the manuscript.

## Abbreviations

AGHB: Alginate–gelatin hydrogel beads
ATSB: Attractive toxic sugar
GC–MS: Gas chromatography–mass spectrometry
VOCs: Volatile organic compounds
SIT: Sterile insect technique
PI: Preference index
Na-Alg: Sodium alginate
CaCl_2_: Calcium chloride
