# Supplementary material for: Evaluation of fruit combinations as potential liquid attractants for hydrogel bait applications targeting Aedes mosquitoes
Source: Parasit Vectors. 2026 Jan 5;19:69. doi: 10.1186/s13071-025-07204-0 (PMC12870405; doi:10.1186/s13071-025-07204-0)
Supplement: Supplementary file 1 — Additional file 1: Fig. S1. Procedure for preparation of fruits solution. Fig. S2. Schematic diagram of the modified Y-olfactometer used to evaluate Aedes mosquito responses to attractants. Fig. S3. Bar graphs showing the mean PI ± SE of Aedes aegypti (males and females) in response to five sets of attractants (a) mango versus sucrose, (b) banana versus sucrose, (c) mango versus banana, (d) mixed versus mango, and (e) mixed versus banana. Positive PI values indicate attraction, whereas negative PI values indicate repellency. Fig. S4. Bar graphs showing the mean PI ± SE of Aedes albopictus (males and females) in response to five sets of attractants (a) mango versus sucrose, (b) banana versus sucrose, (c) mango versus banana, (d) mixed versus mango, and (e) mixed versus banana. Positive PI values indicate attraction, whereas negative PI values indicate repellency. [file 13071_2025_7204_MOESM1_ESM.docx]

**Supplementary information**

**Additional file 1: Fig. S1. Procedure for** preparing the fruits solution.


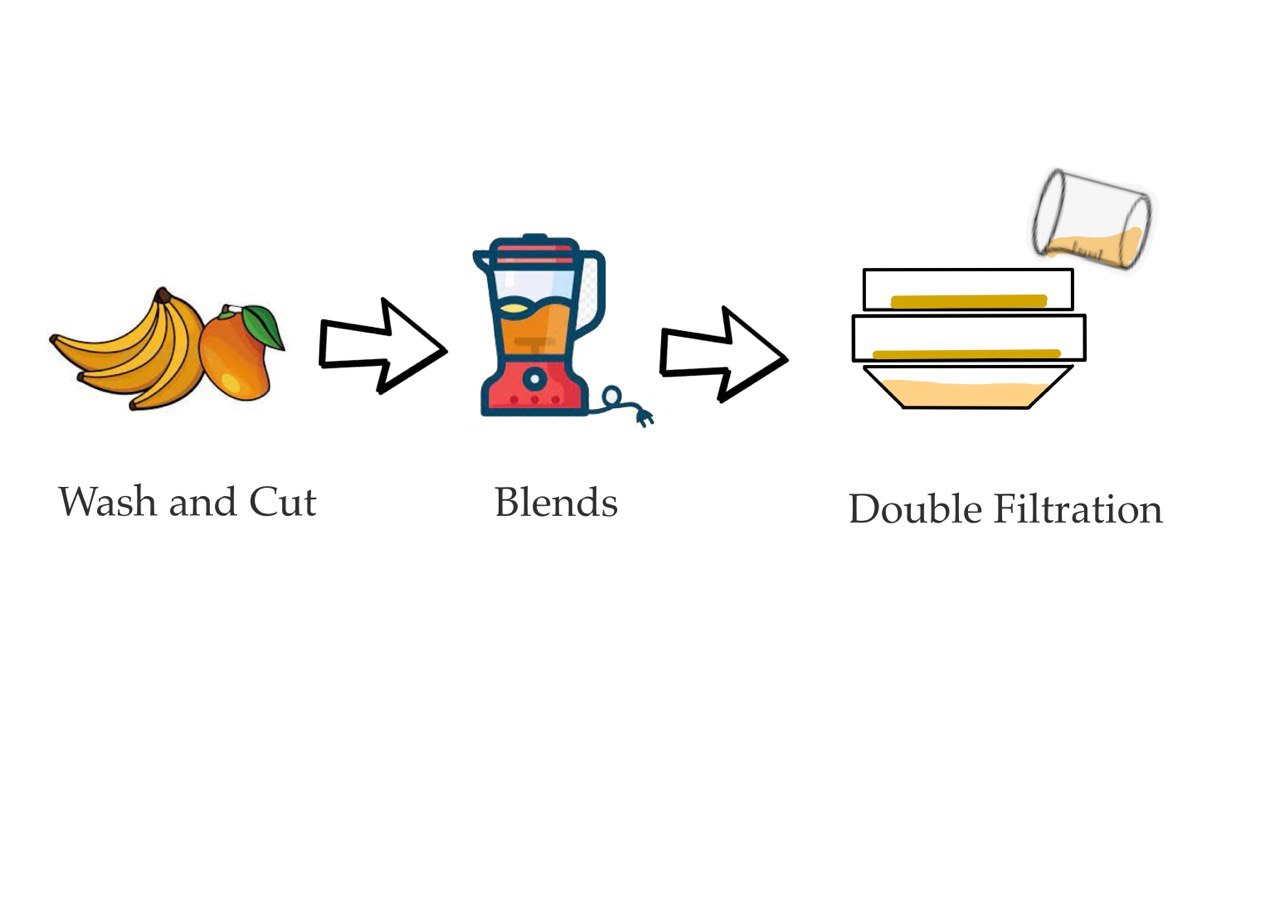


**Fig. S2.** Schematic diagram of the modified Y-olfactometer used to evaluate *Aedes* mosquito responses to attractants.


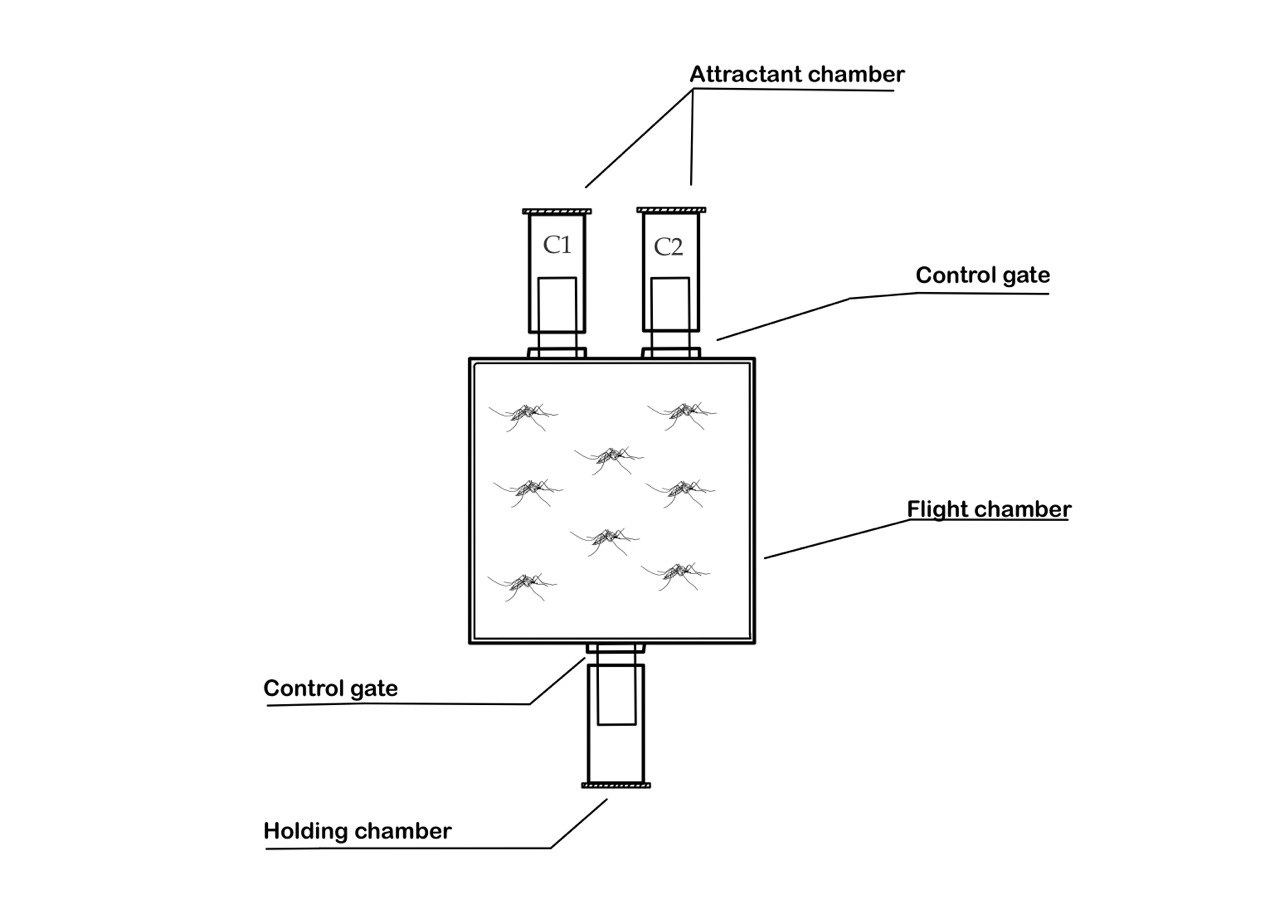


| **Fig. S3**. Bar graphs showing the mean PI ± SE of *Aedes aegypti* (males and females) in response to five sets of attractants (a) mango vs. sucrose, (b) banana vs. sucrose, (c) mango vs. banana, (d) mixed vs. mango, and (e) mixed vs. banana. Positive PI values indicate attraction, whereas negative PI values indicate repellency. |
| --- |

**Fig. S4**: Bar graphs showing the mean PI ± SE of *Aedes albopictus* (males and females) in response to five sets of attractants (a) mango vs. sucrose, (b) banana vs. sucrose, (c) mango vs. banana, (d) mixed vs. mango, and (e) mixed vs. banana. Positive PI values indicate attraction, whereas negative PI values indicate repellency.
