# Supplementary material for: Evaluation of fruit combinations as potential liquid attractants for hydrogel bait applications targeting Aedes mosquitoes
Source: Parasit Vectors. 2026 Jan 5;19:69. doi: 10.1186/s13071-025-07204-0 (PMC12870405; doi:10.1186/s13071-025-07204-0)
Supplement: Supplementary file 2 — Additional file 2: Table S1. Single and mixed attractants for mosquito’s preference tests. Table S2. Mean PI ± SE of male and female Aedes aegypti in response to five sets of attractants based. Table S3. Mean PI ± SE of male and female Aedes albopictus in response to five sets of attractants. Table S4. Volatile organic compounds (VOCs) identified in the mango and banana attractant mixtures. [file 13071_2025_7204_MOESM2_ESM.docx]

**Additional file 2: Table S1.** Single and mixed attractants for mosquito preference tests.

| Set | Attractant Pairs | |
| --- | --- | --- |
|  | Chamber 1 | Chamber 2 |
| A | Mango | Sucrose |
| B | Banana | Sucrose |
| C | Mango | Banana |
| D | Mixed | Mango |
| E | Mixed | Banana |

**Table S2.** Mean PI ± SE of male and female *Aedes aegypti* in response to five sets of attractants.

| Set | Mean PI ± SE of *Ae. aegypti* | | | | |
| --- | --- | --- | --- | --- | --- |
|  | Male | |  | Female | |
|  | Chamber 1 | Chamber 2 |  | Chamber 1 | Chamber 2 |
| A | 0.48 ± 0.20 | -0.48 ± 0.20 |  | 1.00 ± 0.00 | -1.00 ± 0.00 |
| B | 0.92 ± 0.08 | -0.92 ± 0.08 |  | 0.50 ± 0.29 | -0.50 ± 0.29 |
| C | -0.14 ± 0.19 | 0.14 ± 0.19 |  | 0.25 ± 0.43 | -0.25 ± 0.43 |
| D | 0.67 ± 0.19 | -0.67 ± 0.19 |  | 0.30 ± 0.24 | -0.30 ± 0.24 |
| E | 0.43 ± 0.25 | -0.43 ± 0.25 |  | 0.38 ± 0.24 | -0.38 ± 0.24 |

**Table 3.** Mean PI ± SE of male and female *Aedes albopictus* in response to five sets of attractants.

| Set | Mean PI ± SE of *Ae.albopictus* | | | | |
| --- | --- | --- | --- | --- | --- |
|  | Male | |  | Female | |
|  | Chamber 1 | Chamber 2 |  | Chamber 1 | Chamber 2 |
| A | 0.58 ± 0.25 | -0.58 ± 0.25 |  | 0.88 ± 0.13 | -0.88 ± 0.13 |
| B | 0.88 ± 0.13 | -0.88 ± 0.13 |  | 0.38 ± 0.24 | -0.38 ± 0.24 |
| C | -0.08 ± 0.42 | 0.08 ± 0.42 |  | 0.50 ± 0.50 | -0.50 ± 0.50 |
| D | 0.75 ± 0.25 | -0.75 ± 0.25 |  | 0.53 ± 0.18 | -0.53 ± 0.18 |
| E | 0.25 ± 0.28 | -0.25 ± 0.28 |  | 0.58 ± 0.25 | -0.58 ± 0.25 |

**Table S4:** Volatile organic compounds (VOC) identified in the mango and banana attractant mixtures

| **Attractant** | **Compounds** | **Retention time** | **Retention index** | **mV** | **Area (%)** | **References** |
| --- | --- | --- | --- | --- | --- | --- |
| Mango | (Z) 3-hexen-1-ol | 5.75 | 868 | 100 | 0.19 | ^(6, 14)^ |
|  | Ethanol | 1.81 | 465 | 46 | 9.87 | ^(18, 6)^ |
|  | Hexanal | 4.32 | 801 | 100 | 0.17 | ^(14,11)^ |
|  | α-pinene | 12.13 | 948 | 136 | 0.42 | ^(18, 6)^ |
|  | ß-trans-ocimene | 12.13 | 976 | 136 | 0.42 | ^(18)^ |
|  | Caryophyllene | 29.82 | 1494 | 204 | 0.13 | ^(18, 6)^ |
|  | Thujone | 29.99 | 1062 | 152 | 0.16 | ^(16, 14)^ |
|  | α-humule | 31.31 | 1454 | 204 | 0.15 | ^(6, 18)^ |
|  | σ-cadinene | 32.50 | 1512 | 204 | 0.11 | ^(19; 6)^ |
| Banana | Ethyl acetate | 2.23 | 586 | 88 | 11.24 | ^(19, 11)^ |
|  | Hexanal | 4.32 | 801 | 100 | 0.17 | ^(18, 14)^ |
|  | Isoamyl acetate | 6.21 | 820 | 130 | 9.06 | ^(19,20)^ |
|  | Propanoic acid, 2-Methyl | 13.04 | 955 | 144 | 8.74 | ^(21)^ |
|  | Hexanoic acid | 22.01 | 1218 | 186 | 0.32 | ^(21, 22; 23)^ |
|  | Butyl acetate | 3.86 | 785 | 116 | 8.57 | ^(24)^ |
